# Association of accelerometer-measured physical activity and sedentary behavior with unplanned hospitalization in older adults: a 6-year longitudinal study

**DOI:** 10.1007/s11357-025-01756-w

**Published:** 2025-07-12

**Authors:** Joan Ars, Giorgi Beridze, Pau Farrés-Godayol, Laura M. Pérez, Marco Inzitari, Amaia Calderón-Larrañaga, Anna-Karin Welmer

**Affiliations:** 1 Aging Research Center, Department of Neurobiology, Care Sciences and Society (NVS), Karolinska Institutet and Stockholm University, Widerströmska Huset, Tomtebodavägen 18 A, 171 65 Solna, Stockholm, Sweden; 2 RE-FiT Barcelona Research group. Vall d’Hebron Institute of Research (VHIR) and Parc Sanitari Pere Virgili, Barcelona, Spain; 3 Research group on Methodology, Methods, Models, and Outcomes of Health and Social Sciences (M3O), Faculty of Health Sciences and Welfare, University of Vic-Central University of Catalonia (UVic-UCC), Vic, Spain; 4 Faculty of Health Sciences, Universitat Oberta de Catalunya (UOC), Barcelona, Spain; 5 Stockholm Gerontology Research Center, Stockholm, Sweden; 6 Division of Physiotherapy, Department of Neurobiology, Care Sciences and Society, Karolinska Institutet, Stockholm, Sweden; 7 Women´S Health and Allied Health Professionals Theme, Medical Unit Medical Psychology, Karolinska University Hospital, Stockholm, Sweden

**Keywords:** Physical activity, Sedentary behavior, Older adults, Hospitalization

## Abstract

Regular physical activity (PA) and reduced sedentary behavior (SB) are well-established factors in promoting health and lowering the risk of chronic diseases. However, their relationship with unplanned hospitalizations remains unclear. Our aim was to investigate the associations between PA and SB parameters and the risk of unplanned hospital admissions, and the length of hospital stays in older adults. We analyzed data from 657 older adults aged ≥ 66 years from the Swedish SNAC-K study (2016–2018). The ActivPAL3 accelerometer was used to assess PA and SB. Multi-adjusted Cox and Laplace regressions were applied to examine PA and SB in relation to 6-year unplanned hospitalizations. Negative Binomial Regression models were employed to examine their associations with the length of hospital stays. For each 1000 steps/day, 100 low-intensity PA (LPA) walking events, and 15 min/day of time spent in moderate-to-vigorous PA (MVPA), the risk of unplanned admissions decreased (hazard ratio [HR] 0.95, 95% confidence interval [CI] 0.91–0.99, HR 0.77, 95%CI 0.61–0.96, and HR 0.91, 95%CI 0.83–1.00, respectively). Higher step counts and MVPA were also associated with shorter hospital stays (IRR per 1,000 steps: 0.91, 95% CI: 0.86–0.96; IRR per 15 min MVPA: 0.85, 95% CI: 0.75–0.96). No significant associations were found for SB or walking events with hospital stay in fully adjusted models. Our results suggest that higher step counts and greater participation in MVPA are associated with a reduced risk of hospital admissions and shorter hospital stays in older adults. Additionally, a greater number of LPA walking events was linked to lower risk of hospital admissions. These results support the promotion of regular walking and frequent PA as strategies to reduce healthcare burden in aging populations.

**Supplementary Information:**

The online version contains supplementary material available at 10.1007/s11357-025-01756-w.

## Introduction

Hospitalizations among older adults are a significant concern due to their high societal costs and the increased risk of adverse outcomes, which often lead to long-term health complications [1, 2]. Despite the growing importance of this issue, limited research has explored the associations between physical activity (PA), sedentary behavior (SB), and the risk of unplanned hospitalization or the length of hospital stays in older adults [3, 4]. PA interventions have demonstrated benefits in reducing the risk of falls, chronic diseases, functional and cognitive decline, and mortality in older adults [5–7]. In contrast, excessive SB—defined as any waking behavior with an energy expenditure ≤ 1.5 metabolic equivalents (METs) while sitting, lying, or reclining [8]—has been linked to adverse health outcomes independent of PA levels [9]. While PA generally beneficial, it is important to consider that is relationship with hospitalization outcomes may differ across subgroups. For instance, age and mobility limitations might modify the association between PA and hospitalization risk. However, limited evidence exists regarding such potential modifying effects [10]. Accelerometers are the most reliable and precise tools for measuring PA and SB [11, 12]. These devices meticulously track all movements and positional changes throughout the day [13, 14]. Additionally, while accelerometry provides critical information, much of the existing research has focused on the mean daily time spent in different postures and activities during the observation period [3, 15, 16]. Recently, there has been an increased focus on understanding the duration and intensity of stepping sequences, enabling researchers to quantify the cadence of each stepping bout [17, 18]. This event-based perspective allows for a more detailed analysis of postural patterns, identifying changes and sequences. It also provides valuable insights into characterizing behavioral patterns associated with higher daily PA levels [18, 19]. This improves research on the health benefits of increased PA and reduced SB, supporting public health messaging.

This study aimed to examine the associations between PA and SB parameters, measured using accelerometry, and the risk of unplanned hospital admission and the number of hospitalization days in a cohort of community-dwelling older adults.

## Methods

### SNAC-K and data collection

The Swedish National Study on Aging and Care in Kungsholmen (SNAC-K) is an ongoing cohort study that aims to increase the understanding of the aging process and identify preventive strategies for improved health and care for older adults [20]. Information is collected through interviews, clinical examinations, and tests by trained nurses, psychologists, and physicians. At baseline (2001–2004), a random sample of 3363 individuals (73.3% participation rate) was selected from each of 11 age groups (60, 66, 72, 78, 81, 84, 87, 90, 93, 96, and ≥ 99 years). For younger cohorts (60–78 years), follow-up examinations are performed every six years, and for older cohorts (78 + years), every 3 years [21]. In the sixth wave of follow-up, between 2016 and 2018, an objective assessment of PA and SB using ActivPAL3 *(PAL Technologies Ltd., Glasgow, UK)* accelerometers was introduced into the study [22, 23]. Of 1310 participants in the age groups 66, 81, 84, 87, 90, 93, and ≥ 96 years examined during this period, 684 were considered eligible (excluding those with severe cognitive impairment or inability to move indoors without assistance) and agreed to wear the accelerometer for seven consecutive days. From this group, 27 participants were excluded for the following reasons: they did not wear the device according to instructions (*n* = 2), they had fewer than four valid days of data (*n* = 17), or they did not consent to be followed in registers (*n* = 8), leaving an analytical sample of 657 people (*see the flowchart of included and excluded participants in *supplementary material part 1).

### Physical activity and sedentary behavior assessment

The thigh-worn ActivPAL3 accelerometer was used to assess PA and SB. This device uses accelerometer-derived information about thigh position to determine the start and end of each period spent sitting/lying, standing, walking, step counts, and postural transitions [13]. Participants were asked to continue their usual daily habits while wearing the ActivPAL3 for seven consecutive days during all waking hours (excluding showering and swimming activities), starting the day after the SNAC-K study visit. We considered valid assessments from subjects who wore the accelerometer for at least four consecutive days (24-h blocks) and whose wear time was at least 10 h during waking hours. We used 24-h blocks (midnight to midnight) for the data extraction, excluding half days based on previously described recommendations [24]. The Excel macro-HSC PAL 2.21 analysis software developed by Dr Philippa Dall and Prof Malcolm Granat, Faculty of Health and Life Sciences, Glasgow Caledonian University [25], was used to extract the data from the devices. The software utilized second-by-second epochs to classify posture based on inclination and to determine the duration and number of steps taken; this approach captures every daily movement, increasing the robustness and reliability of measurements [26]. We used the CREA and VANE algorithms from the ActivPAL3 software manufacturer (PAL Analysis version 8, PAL Technologies Ltd, Glasgow, Scotland) [27] to extract the data regarding the non-wear-time to set the HSC software for the data extraction. The HSC software combines individual walking steps (second-by-second) into a single event and classifies the intensity of this event based on cadence. For the PA variables, we assessed [1] the number of steps (step count); [2] the time spent in low-intensity PA (LPA) (in minutes), determined by standing and walking time, the walking periods having a cadence of < 100 steps/min ^28^); [3] the time in moderate-to-vigorous intensity PA (MVPA) in minutes, determined using walking periods a cadence of ≥  = 100 steps/min ^28^); [4] the number of LPA walking events and the number of MVPA walking events [5]. LPA and MVPA walking events represent continuous periods of walking, excluding standing. Each walking event is subsequently categorized as MVPA or not based on the average cadence of the steps [19]. Walking events were derived from second-by-second activPAL output, allowing for accurate cadence calculation across each event. Cadence has been validated as an effective and precise proxy for estimating walking intensity, previously suggested as a reliable method for defining physical activity intensity [28, 29]. For the SB variable, we assessed the total time in hours in SB, which was classified as the total time in a sitting or lying position. (see supplementary material, part 2, *for additional details on accelerometer configuration and how data was extracted).*

### Outcome definition

During the follow-up, hospitalization data were obtained through the National Patient Register, including information from inpatient care and specialized outpatient care for all subjects throughout Sweden [30]. Participants’ vital status was extracted from the Swedish Cause of Death Register. Two distinct outcomes were derived from the inpatient data over a 6-year period following the baseline accelerometer assessment conducted as a part of the SNAC-K visit: [1] the time to first unplanned hospital admission and [2] the total number of hospital days attributable to unplanned admissions during the follow-up [31].

### Covariates

During the interviews, data on age, sex, cohabitation status, body mass index (BMI), and the highest level of education attained (elementary, high school, or university and above) were collected. BMI was calculated based on weight and height using standard methods adapted for older adults [32]. Mobility limitations were assessed using the five times sit-to-stand test (5 STS). The 5 STS was conducted by asking the participants to stand up and sit down five times as fast as they could, without using their arms, and categorized their ability to perform five consecutive chair stands (yes/no) [33]. Studies show it is highly reliable in healthy adults and those with pathologies [34]. Multimorbidity burden was operationalized as the number of chronic diseases based on a previously described methodology [35]. Patients hospitalized within the 3 years prior to their baseline SNAC-K examination were categorized as having previous hospitalizations.

### Statistical analysis

Categorical variables were presented as frequencies and percentages, whereas continuous variables were described using the mean and standard deviation. Exposures were presented as the daily number of steps (per 1000), daily time spent on LPA or SB (per hour), daily time spent on MVPA (per 15 min), daily LPA walking events (per 100), and daily MVPA walking events (per 10). Different denominators were established for each exposure to achieve clinically meaningful estimates. The median value was selected as the cut-off for the variables LPA and MVPA walking events due to the limited literature exploring these variables. We analyzed the associations between PA or SB and the time until the first unplanned hospitalization using Cox proportional hazards models and the derived hazard ratios (HR) with 95% confidence intervals (CIs). Participants who died before 31.12.2021 without any unplanned hospitalization during the study period were censored at the date of death. Those who did not experience hospitalization or death were censored at the end of follow-up on 31.12.2021. We used Schoenfeld residuals to assess the assumption of proportional hazards, and no evidence of violation was detected. In addition to the Cox models, we employed Laplace regression models to estimate the association between exposures and the median time to the first unplanned hospitalization. To evaluate potential heterogeneity by age group and mobility limitation, we tested the multiplicative interactions between age and PA variables, and between mobility limitation and the PA variables, in relation to both outcomes. A stratified analysis was planned if the interaction term had a *p*-value < 0.10.

For the subgroup of participants with at least one unplanned hospitalization during follow-up, we employed negative binomial regression models to estimate the Incidence Rate Ratios (IRR) and 95% CIs for the associations between PA or SB and the number of hospital days. An offset variable was used to account for each subject’s observation period (person-days).

For each exposure, we performed two models adjusting for potential confounders in a stepwise manner. Model 1 was adjusted for age, sex, and education. In Model 2, we additionally adjusted for BMI, cohabitation status, multimorbidity burden, the 5 STS, and previous hospitalizations. Furthermore, for the LPA and MVPA walking event exposures, we additionally considered the potential confounding effect of total walking time in LPA and MVPA, respectively, to see the potential influence of behavioral patterns independently of the total time spent in each PA intensity. Data were analyzed using Stata 18.0 (Stata-Corp LP, College Station, TX, USA).

## Results

Among the 657 participants, the mean age at the time of the accelerometer assessment was 73.4 (SD: 9.0) years, and 64.1% were female. More than half had a university-level education (57.5%) and were married or living with someone (55.9%). Participants had a mean BMI of 25.7 (3.9) and an average of 4.9 (2.9) chronic diseases. On average, participants wore the accelerometer for 14.4 (1.1) hours per day and took 8,654 (3,756.6) steps per day (Table 1). During the follow-up period, 283 participants (43.1%) experienced at least one unplanned hospitalization, with a mean duration of 14.5 days (SD: 19.9). Participants who were excluded from the study were, on average, older than those included (see Supplementary Material, Part 3).

**Table 1:** Baseline characteristics of the study population

Characteristics	(*n* = 657)
Age	73.4 (9)
Women	421 (64.1)
Educational level	
Elementary or High school	279 (42.5)
University	378 (57.5)
Cohabitation status	
Living alone	290 (44.1)
Married/Living together	367 (55.9)
Body Mass Index	25.7 (3.9)
Chronic diseases, total number	4.9 (2.9)
5 STS, seconds	16.5 (19.4)
Daily wear time, hours	14.4 (1.1)
Daily number of steps	8654 (3757)
Daily time spent on SB, hours	8.8 (1.6)
Daily time spent on LPA, minutes	307.8 (92.2)
Daily time spent on MVPA, minutes	31.3 (25.8)
Daily LPA walking events	323 (104)
Daily MVPA walking events	41 (27)

Data are presented as means (SD) for continuous variables and n (%) for categorical variables. 5 STS = five sit-to-stand; *SB* sedentary behavior, *MVPA* moderate-vigorous physical activity, *LPA* low physical activity

**Fig. 1:**
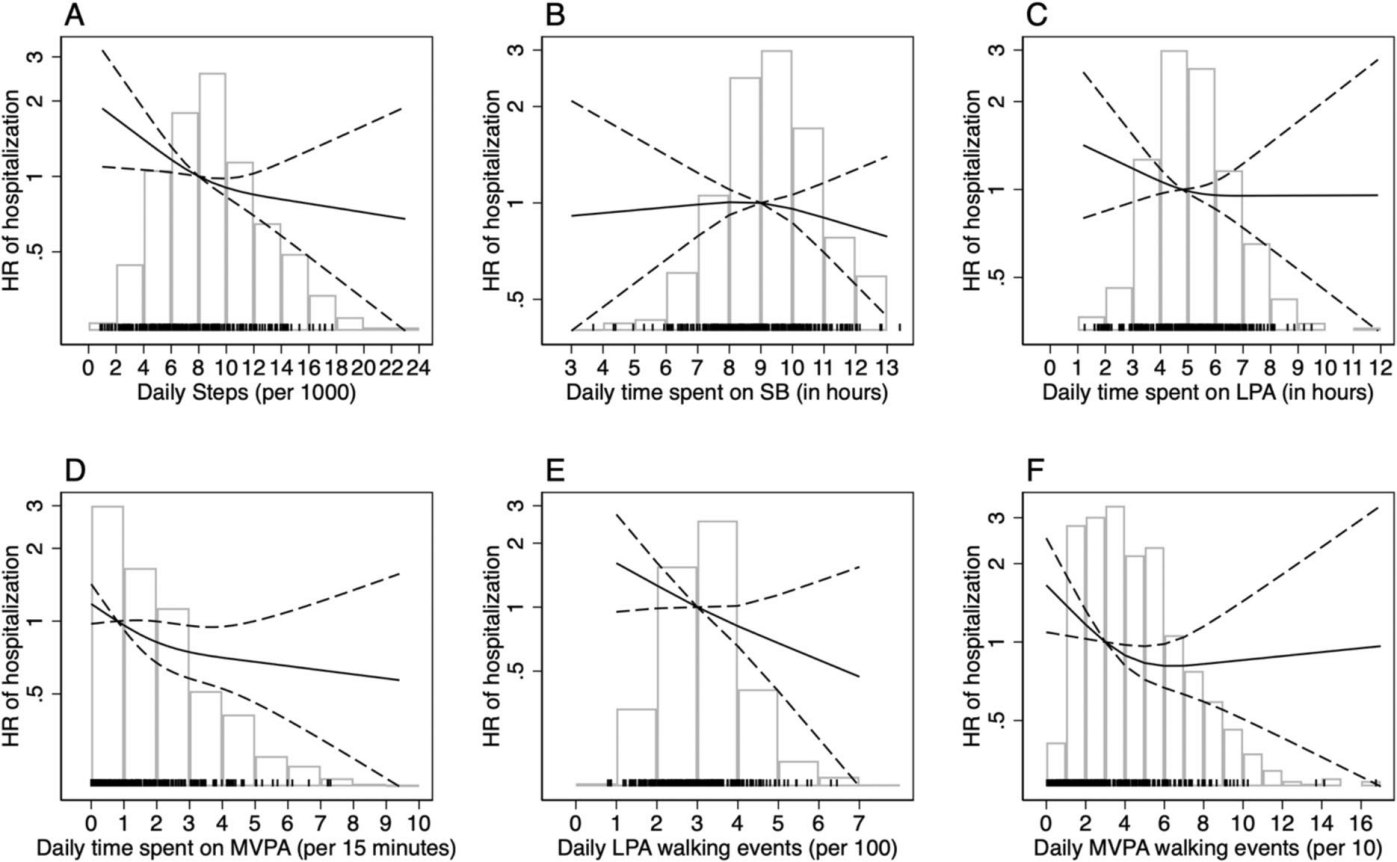
Associations between steps (Panel **A**), SB (Panel **B**), LPA (Panel **C**), MVPA (Panel **D**), LPA walking events (Panel **E**), MVPA walking events (Panel **F**), and the risk of unplanned hospitalization

The Cox model revealed that for each additional 1,000 steps/day and 100 LPA, and 15 min/day of MVPA, the risk of unplanned admissions decreased significantly in both Model 1 and Model 2 (Model 2: HR 0.95, 95% CI 0.91–0.99; HR 0.76, 95% CI 0.60–0.95; HR 0.94, 95% CI 0.89–1 and HR 0.90, 95% CI 0.82–0.99) (Table 2 and Fig. 1). In contrast, no significant associations were observed for SB, LPA, and MVPA walking events. In the Laplace model 2, for each additional 1,000 steps/day, the median time to first admission was postponed by 42.8 (95% CI 0.25–85.3) days. No significant interactions were found between mobility limitations and age and, exposures in relation to the time until the first unplanned hospitalization (*p*-value for mobility limitations interaction: steps 0.889; SB 0.245; LPA 0.903; LPA walking events 0.722; MVPA 0.112; MVPA walking events; 0.253) and (*p*-value for age interaction; steps 0.920; SB 0.912; LPA 0.796; LPA walking events 0.345; MVPA 0.707; MVPA walking events 0.158).

**Table 2:** Hazard ratios (HRs) and 95% confidence intervals (CIs) for 6-year unplanned hospitalizations and median difference in the number of days to first hospitalization

	Cox regression, HR (95% CI) for first hospitalization		Laplace regression, median difference (95% CI) in number of days to first hospitalization	
	Model 1	Model 2	Model 1	Model 2
Daily steps, per 1,000	0.92 (0.88, 0.95)	0.95 (0.91, 0.99)	78.02 (38.01, 118.03)	42.76 (0.25, 85.28)
Daily time spent on SB, per hour	1.04 (0.96, 1.12)	0.99 (0.91, 1.06)	−8.68 (−101.32, 83.96)	0.32 (−77.03, 77.66)
Daily time spent on LPA, per hour	0.91 (0.84, 0.99)	0.95 (0.87, 1.04)	65.50 (−31.12, 162.12)	48.04 (−12.53, 108.61)
Daily LPA walking events, per 100	0.69 (0.56, 0.86)	0.76 (0.60, 0.95)	286.48 (56.57, 516.39)	191.85 (−91.76, 475.46)
Daily time spent on MVPA, per 15 min	0.83 (0.76, 0.91)	0.90 (0.82, 0.99)	169.49 (60.70, 278.27)	91.90 (−8.09, 191.91)
Daily MVPA walking events, per 10	0.94 (0.88, 1.02)	0.94 (0.89, 1)	64.89 (−0.39, 130.18)	56.68 (−10.25, 123.60)

Abbreviations: *SB* sedentary behavior, *MVPA* moderate-vigorous physical activity, *LPA* low physical activity. Note: The total number of subjects included in the models was: Model 1: 657; Model 2: 651. Model 1 is adjusted for age, sex, and education, and Model 2 is additionally adjusted by BMI, cohabitation status, multimorbidity burden, 5 STS, and prior hospitalizations. For the LPA and MVPA walking events exposures, all models are also adjusted by total walking time on low and moderate-vigorous intensity, respectively

Figure 1 illustrates the adjusted associations between various PA and SB parameters and the risk of unplanned hospitalization, based on Model 2 using restricted cubic splines. A clear inverse relationship was observed between daily step count and hospitalization risk, with the hazard ratio (HR) decreasing progressively as the number of steps increased (Panel A). Similarly, greater time spent in MVPA was associated with a markedly lower risk of hospitalization, particularly up to approximately 60 min per day (Panel D). In contrast, no consistent associations were identified for sedentary time (Panel B) or total time spent in LPA (Panel C), with HR curves remaining close to the null value and wide confidence intervals. Notably, the number of LPA walking events (Panel E) was significantly associated with reduced hospitalization risk. MVPA walking events (Panel F) showed a non-linear relationship with hospitalization risk, though the association was weaker and less consistent than those observed for total MVPA time and step count.

The Negative Binomial regression models indicated that each additional 1,000 steps/day and each additional 15 min/day of MVPA were significantly associated with shorter hospital stays (Model 2: IRR 0.91, 95% CI: 0.86–0.96; IRR 0.85, 95% CI: 0.75–0.96, respectively). In contrast, no significant associations were observed for SB, LPA, or the number of LPA or MVPA walking events. We found significant interactions between age and MVPA walking events in relation to the number of hospitalization days (*p*-value for interaction: 0.085). In stratified analyses by age group, MVPA walking events were significantly associated with fewer hospitalization days in the oldest-old under Model 1; however, this association was attenuated and no longer significant after full adjustment in Model 2 (see Supplementary material, part 4). No significant interactions were found between mobility limitations and age and, exposures in relation to the time until the first unplanned hospitalization (*p*-value for mobility limitations interaction: steps 0.754; SB 0.414; LPA 0.144; LPA walking events 0.711; MVPA 0.894; MVPA walking events; 0.985) and (*p*-value for age interaction; steps 0.602; SB 0.324; LPA 0.533; LPA walking events 0.139; MVPA 0.559) (Table 3).

**Table 3:** Incidence rate ratios (IRR) and 95% confidence intervals (CIs) for total number of hospitalization days

	Model 1	Model 2
Daily steps, per 1000	0.91 (0.86, 0.96)	0.91 (0.86, 0.96)
Daily time spent on SB, per hour	1.09 (0.98, 1.20)	1.07 (0.97, 1.18)
Daily time spent on LPA, per hour	0.98 (0.87, 1.09)	0.99 (0.88, 1.12)
Daily LPA walking events, per 100	0.99 (0.77, 1.27)	1.03 (0.79, 1.34)
Daily time spent on MVPA, per 15 min	0.83 (0.74, 0.94)	0.85 (0.75, 0.96)
Daily MVPA walking events, per 10	0.98 (0.91, 1.07)	1.02 (0.93, 1.11)

Abbreviations: *SB* sedentary behavior, *MVPA* moderate-vigorous physical activity, *LPA* low physical activity. Note: The total number of subjects included in the models was: Model 1: 280; Model 2: 279. Model 1 is adjusted for age, sex, and education, and Model 2 is additionally adjusted by BMI, cohabitation status, multimorbidity burden, 5 STS, and prior hospitalizations. For the LPA and MVPA walking events exposures, all models are also adjusted by total walking time on low and moderate-vigorous intensity, respectively

## Discussion

In this longitudinal study, we found that older adults who took more daily steps, spent more time in MVPA, and accumulated more LPA walking events had a reduced risk of unplanned hospital admissions. Additionally, a higher number of steps and more time spent in MVPA were associated with fewer days spent in the hospital. In contrast, no significant associations were observed for SB, LPA, or the number of LPA or MVPA walking events. No consistent modifying effects were observed for age or mobility limitations with the exposures.

Consistent with previous studies, we found that higher PA levels were associated with a lower risk of hospital admission, mainly when performed at moderate-to-vigorous intensities [3, 4]. A study based on data from a Swedish cohort, including adults with a mean age of 45 years, followed up for 15 years, also supported the idea that more PA reduces the risk of hospitalization [4]. Unlike that study, we found no association between SB and the risk of hospital admission. Differences in age and baseline activity levels between samples may partly explain these discrepancies. Prior research suggests that SB may exert stronger negative effects in younger than in older populations [36]. Although further research is needed, these findings support the relevance of moving beyond total PA time toward more focus on stepping pattern profiles when studying health outcomes in older adults. Current evidence suggests that spending shorter bouts on SB is the key to reducing its negative consequences [15, 37, 38]. It is plausible that individuals who participate in more walking events also tend to engage in shorter bouts of SB, potentially explaining why we did not find significant associations with SB and hospital admission in our study. This aligns with our results, as those who spent more time on MVPA and performed more steps also showed a shorter length of hospital stay. Our study also introduces novel variables such as the number of PA walking events, which had not previously been evaluated in large cohort studies of older adults. These event-based metrics provide an alternative way of characterizing activity behavior and may help identify patterns associated with hospital-related outcomes.

While our study does not delve into the physiological mechanisms as done in Watts et al.’s paper [3], their investigation sheds light on several potential pathways linking PA to reduced hospitalization risk among older adults. The leading causes of hospitalization in this age group include cardiovascular diseases, respiratory diseases, sepsis, osteoarthritis, and other somatic conditions [3, 39–41]. Thus, potential mechanisms may include improved cardiovascular health [15, 36], reduced risk of conditions such as venous thromboembolism and ischemic stroke, enhanced immune competency [42, 43] that may reduce the risk of infections such as urinary tract infections, reduced systemic inflammation [44, 45], improved respiratory health [46, 47] potentially reducing the risk of respiratory infections like pneumonia, and through these mechanisms protection against the risk of all-cause mortality [15, 48, 49]. Frailty and sarcopenia, which are associated with longer hospital stays [50–55], can be prevented by regular PA [56, 57]. While evidence regarding recommendations for PA in older adults is available, there is also evidence indicating differences in the dosage and intensity of PA among various older population profiles [58, 59]. However, strategies such as higher volume and intensity of PA, as well as shorter episodes of SB, have shown promise [5, 9, 49, 60, 61]. Incorporating PA as an exercise snack, defined as brief intermittent bouts [62], throughout the day has gained traction due to its ease of implementation in various settings and among populations with diverse health conditions [62–65]. Although exploratory studies have suggested that movement patterns such as frequent walking events may relate differently to hospitalization depending on functional status, our study did not identify significant interaction effects with mobility limitations. Future research may help clarify whether certain subgroups of older adults could benefit from tailored PA recommendations based on mobility or frailty profiles.

This study has several strengths. The primary strength lies in the utilization of accelerometry data from a large number of older adults living in the community. The accelerometry data extraction process was robust and transparent [13, 66], minimizing potential measurement bias. Linking the SNAC-K cohort to reliable national inpatient data also enabled longitudinal investigation. The study also has limitations. Although we controlled for several potential confounders including prior hospitalization, we cannot exclude the possibility of residual confounding or reverse causality. The study sample consists of Swedish older adults living in an urban area with a high socio-economic status. The sample had a high level of physical activity. Moreover, individuals with mobility limitations were underrepresented, which may have influenced the observed associations. As a result, the estimates may not be generalizable to other populations. Furthermore, only cognitively healthy people were included in the sample. Therefore, our study does not cover the whole spectrum of cognitive capacity. Additionally, while the National Patient Register includes diagnostic codes for hospitalizations, we did not have information data on the causes of admission, which limited our ability to conduct detailed analyses on the specific reasons for hospitalization.

## Conclusion

Our findings suggest that greater participation in PA, particularly through higher step counts and more time spent in MVPA, is associated with a lower risk of unplanned hospital admission and fewer hospital days in older adults. These associations support current recommendations promoting MVPA in later life. Although we did not observe significant associations between LPA walking events and the duration of hospital stay, the consistent trend toward benefit reinforces the potential of promoting lighter, more frequent movement throughout the day. Importantly, no significant interaction effects with mobility limitations were found, indicating that these associations may be relevant across a range of functional capacities. These results underscore the value of simple, scalable PA interventions in reducing healthcare burden and improving outcomes among community-dwelling older adults, while also highlighting the need for further research into tailored strategies for specific subgroups.

## Supplementary Information

Below is the link to the electronic supplementary material.

Supplementary Material 1 (DOCX 118 KB)

## Data Availability

Data are from the SNAC-K project, a population-based study on aging and dementia (http://www.snac-k.se/). Access to these original data is available to the research community upon approval by the SNAC-K data management and maintenance committee. Applications for accessing these data can be submitted via the SNAC-K website (https://www.snac-k.se/for-researchers/application-form/).

## Abbreviations

PA: Physical Activity
SB: Sedentary Behavior
METs: metabolic equivalents
SNAC-K: Swedish National Study on Aging and Care in Kungsholmen
BMI: Body Mass Index
LPA: Low Physical Activity
MVPA: Moderate-to-Vigorous Physical Activity
HR: Hazard Ratio
CIs: confidence intervals
