# Supplementary material for: Association of accelerometer-measured physical activity and sedentary behavior with unplanned hospitalization in older adults: a 6-year longitudinal study
Source: GeroScience. 2025 Jul 12;48(2):2345–55. doi: 10.1007/s11357-025-01756-w (PMC12972329; doi:10.1007/s11357-025-01756-w)
Supplement: Supplementary file 1 — Supplementary Material 1 (DOCX 118 KB) [file 11357_2025_1756_MOESM1_ESM.docx]

**Supplementary Material**

Part 1: Flowchart with included vs excluded participants.


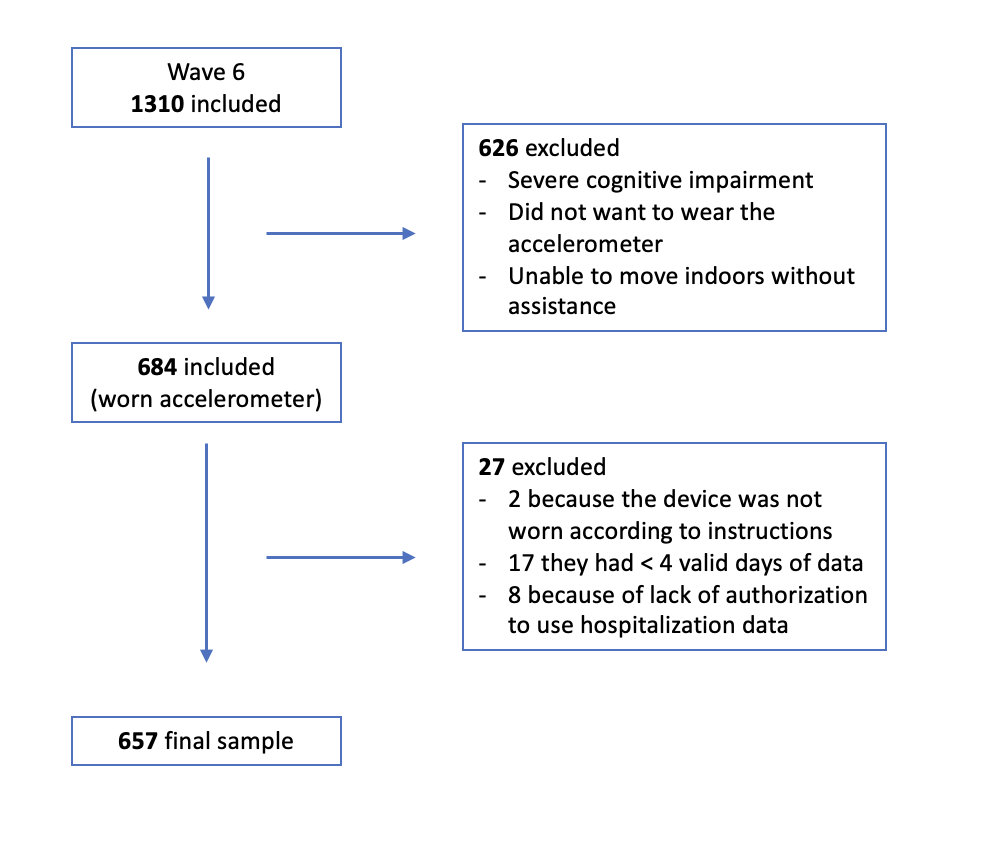


**Part 2: Accelerometer data extraction.**

| Item | Observations |
| --- | --- |
| Monitor version | ActivPAL3. |
| Rationale for selecting ActivPAL monitor | Description of sedentary behaviour and physical activity in a sample of community-dwelling older adults. |
| Reliability (inter- and intra-instrument) for the device selected (if available) | Inter device reliability for the activPAL device ranged from 0.79 to 0.99 (1). |
| Method and location of monitor attachment | Right anterior midline of thigh right dominant with a Micropore 2.5 cm tape for skin. |
| Wear period and number of days | 7 consecutive days during all waking hours (excluding showering and swimming). |
| ActivPAL software version | Version 8.11.8.75 |
| Settings used:   - Minimum sitting period - Minimum upright period | 1 second epoch’s  1 second epoch’s |
| Diary data collected and details collected | Sleep time, shower time and water activities. |
| Goal for the sampling periods observed | At least 10 h of data per day and 4 days of data. |
| Method(s) for estimating wearing time/removing time in bed/sleep | Heat maps of included and excluded data visually checked (side by side) to detect likely misclassification of data to be included/excluded.  Probable errors were cross-checked with the diaries (where available) and with a second expert. |
| What quality control checks were implemented | All subjects were reviewed 1:1 by an expert and in cases where interpretation was difficult, a second expert was consulted. The outputs obtained were quality checked to ensure that all times matched and that there were no errors in the data. |
| Type of action taken when data were determined to be invalid | Data considered invalid were excluded. |
| Compliance criteria to define a valid day | The participants wore the ActivPal for ≥10 h |
| Number of days | 4 consecutive days in blocks of 24h (without using half days) (2). |
| Definition of a day | 24 hours’ time block (midnight to midnight) (2). |
| Data processing package used, and methods used to generate key summary variables | ActivPAL software Version 8.11.8.75 using VANE algorithm to create events files. Excel macro-HSC PAL analysis software 2.21 to extract the data (3). |
| Criteria for defining moderate to vigorous physical activity | Calculate MVPA and LPA (using cadence) directly (using ≥=100 steps/min and <100 steps/min as thresholds for MVPA and LPA (2). |

**Part 3: Baseline characteristics of included vs excluded participants.**

| Characteristics | Excluded  (n=626) | Included  (n=657) | p-value |
| --- | --- | --- | --- |
| Age | 81.4 (10) | 73.4 (9) | <0.001 |
| Women | 399 (63.7%) | 421 (64.1%) | 0.12 |
| Education level | | | |
| Elementary or High school  University | 327 (52.2%)  294 (47.0%) | 279 (42.5%)  378 (57.5%) | <0.001 |
| Cohabitation status, n (%) | | | |
| Living alone  Married/Living together | 367 (58.6%)  233 (37.2%) | 290 (44.1%)  367 (55.9%) | <0.001 |
| Chronic diseases, total number | 6.8 (3.5) | 4.9 (2.9) | <0.001 |
| Five chair-stand test, seconds | 41.8 (31.8) | 16.5 (19.4) | <0.001 |
| MMSE | 25.4 (6.7) | 28.7 (1.3) | <0.001 |

*Note: Data are presented as mean (SD) for continuous measures, and n (%) for categorical measures. MMSE= Mini Metal State Examination.*

**Part 4:** **Incidence rate ratios (IRR) and 95% confidence intervals (CIs) for total number of hospitalization days, stratified by age.**

|  | **Age** $\boldsymbol{<}$**70** | | **Age** $\boldsymbol{\geq}$ **80** | |
| --- | --- | --- | --- | --- |
|  | **Model 1**  **(N=106)** | **Model 2**  **(N=106)** | **Model 1**  **(N=174)** | **Model 2**  **(N=173)** |
| Daily MVPA walking events, per 10 | 1.07  (0.92, 1.26) | 1.10  (0.94, 1.28) | **0.91**  **(0.84, 0.99)** | 0.99  (0.90, 1.08) |

References

1. Grant PM, Ryan CG, Tigbe WW, Granat MH. The validation of a novel activity monitor in the measurement of posture and motion during everyday activities. Br J Sports Med. 2006 Dec 1;40(12):992–7.

2. Farrés-Godayol P, Ruiz-Díaz MÁ, Dall P, Skelton DA, Minobes-Molina E, Jerez-Roig J, et al. Determining minimum number of valid days for accurate estimation of sedentary behaviour and awake-time movement behaviours using the ActivPAL3 in nursing home residents. Eur Rev Aging Phys Act. 2023 Oct 7;20(1):19.

3. Iveson AMJ, Granat MH, Ellis BM, Dall PM. Concurrent Measurement of Global Positioning System and Event-Based Physical Activity Data: A Methodological Framework for Integration. J Meas Phys Behav. 2021 Mar 1;4(1):9–22.

4. Lee LFR, Dall PM. Concurrent agreement between ActiGraphⓇ and activPALⓇ in measuring moderate to vigorous intensity physical activity for adults. Med Eng Phys. 2019 Dec;74:82–8.
